# Supplementary material for: Exploring conformational equilibria of a heterodimeric ABC transporter
Source: eLife. 2017 Jan 4;6:e20236. doi: 10.7554/eLife.20236 (PMC5216877; doi:10.7554/eLife.20236)
Supplement: Figure 5—source data 1. — DOI: http://dx.doi.org/10.7554/eLife.20236.017 [file elife-20236-fig5-data1.docx]

Figure 5-Source data 1. **Parameters of the two-Gaussian fit.**

|  | **Nucleotide** | **r_1_ [nm]** | **σr_1_ [nm]** | **r_2_ [nm]** | **σr_2_ [nm]** | **IF frac [%]^*^** | **OF frac [%]^**^** |
| --- | --- | --- | --- | --- | --- | --- | --- |
| **50^TM287^-271^TM287^ WT** | ATP-Vi-Mg | 2.528 | 0.241 | 3.229 | 0.924 | 6 | 94 |
|  | AMP-PNP-Mg | 2.528 | 0.19 | 3.229 | 0.924 | 85.4 | 14.6 |
|  | ATP-Mg | 2.561 | 0.19 | 3.229 | 0.924 | 36.7 | 63.3 |
|  | ATP-EDTA | 2.542 | 0.197 | 3.229 | 0.924 | 36.9 | 63.1 |
|  | ATPγS-Mg | 2.56 | 0.241 | 3.229 | 0.924 | 45.2 | 54.8 |
|  | ADP-Mg | 2.528 | 0.236 | 3.229 | 0.924 | 90.1 | 9.9 |
| **131^TM288^-248^TM288^ WT** | ATP-Vi-Mg | 1.998 | 0.339 | 3.497 | 1.1 | 37.9 | 62.1 |
|  | AMP-PNP-Mg | 1.998 | 0.339 | 3.222 | 0.889 | 100 | 0 |
|  | ATP-Mg | 1.998 | 0.339 | 3.307 | 1.1 | 56.8 | 43.2 |
|  | ATP-EDTA | 1.998 | 0.339 | 3.596 | 0.695 | 84.5 | 15.5 |
|  | ATPγS-Mg | 1.998 | 0.339 | 3.307 | 0.86 | 72.9 | 27.1 |
|  | ADP-Mg | 1.998 | 0.339 | 3.18 | 0.903 | 100 | 0 |
| **460^TM287^-363^TM288^ WT** | ATP-Vi-Mg | 2.973 | 0.33 | 3.917 | 0.8 | 35 | 65 |
|  | AMP-PNP-Mg | 2.973 | 0.33 | 3.99 | 0.678 | 90.7 | 9.3 |
|  | ATP-Mg | 2.973 | 0.33 | 3.99 | 0.9 | 25.7 | 74.3 |
|  | ATP-EDTA | 2.973 | 0.33 | 3.99 | 0.9 | 80.7 | 19.3 |
|  | ATPγS-Mg | 2.973 | 0.33 | 3.694 | 0.9 | 63.7 | 36.3 |
|  | ADP-Mg | 2.973 | 0.33 | 3.604 | 0.658 | 82 | 18 |
| **50^TM287^-271^TM287^ E517Q** | AMP-PNP-Mg | 2.5 | 0.222 | 3.229 | 0.924 | 64.9 | 35.1 |
|  | ATP-Mg | 2.572 | 0.1 | 3.229 | 0.924 | 5.79 | 94.21 |
|  | ATP-EDTA | 2.572 | 0.166 | 3.229 | 0.924 | 14.1 | 85.9 |
| **131^TM288^-248^TM288^ E517Q** | AMP-PNP-Mg | 3.062 | 0.711 | 3.891 | 0.662 | 100 | 0 |
|  | ATP-Mg | 1.998 | 0.339 | 3.891 | 0.662 | 24.9 | 75.1 |
|  | ATP-EDTA | 1.998 | 0.339 | 3.891 | 0.662 | 31.2 | 68.8 |
| **460^TM287^-363^TM288^ E517Q** | AMP-PNP-Mg | 2.973 | 0.33 | 3.99 | 0.618 | 65.8 | 34.2 |
|  | ATP-Mg | 2.973 | 0.33 | 3.99 | 1.1 | 13 | 87 |
|  | ATP-EDTA | 2.973 | 0.33 | 3.99 | 1.1 | 21.2 | 78.8 |
|  | *The estimated errors in the fractions of the populations are 10%.  **Forcing the fit of broad and non-Gaussian distance distributions with one Gaussian distribution, as in the OF state of the extracellular pair, leads to a large σ (~ 1 nm), which decreases the reliability of the obtained fractions. | | | | | | |
